# Longitudinal stability of retinal blood flow regulation in response to flicker stimulation and systemic hyperoxia in mice assessed with laser speckle flowgraphy

**DOI:** 10.1038/s41598-020-75296-y

**Published:** 2020-11-13

**Authors:** Junya Hanaguri, Harumasa Yokota, Masahisa Watanabe, Lih Kuo, Satoru Yamagami, Taiji Nagaoka

**Affiliations:** 1 Division of Ophthalmology, Department of Visual Sciences, Nihon University School of Medicine, 30-1 Oyaguchi-Kamicho, Itabashi-ku, Tokyo, 173-8610 Japan; 2 Department of Medical Physiology, Texas A&M University Health Science Center, Bryan, TX USA

**Keywords:** Neuro-vascular interactions, Diagnostic markers, Physiology

## Abstract

This study aimed to evaluate longitudinal changes in retinal blood flow in response to flicker stimulation and systemic hyperoxia in mice using a laser speckle flowgraphy (LSFG-Micro). The retinal blood flow in vascular area surrounding the optic nerve head was measured in 8-week-old male mice every 2 weeks until age 20-week. The coefficient of variation of retinal blood flow under resting condition was analyzed every 2 weeks to validate the consistency of the measurement. On day 1 of the experiment, retinal blood flow was assessed every 20 s for 6 min during and after 3 min flicker light (12 Hz) stimulation; on day 2, retinal blood flow was measured every minute for 20 min during and after 10 min systemic hyperoxia; and on day 3, electroretinography (ERG) was performed. Body weight, systemic blood pressure, and ocular perfusion pressure increased significantly with age, but the resting retinal blood flow and ERG parameters remained unchanged. Retinal blood flow significantly increased with flicker stimulation and decreased with systemic hyperoxia, independent of age. The LSFG-Micro provides consistent and reproducible retinal blood flow measurement in adult mice. Longitudinal assessments of retinal blood flow in response to flicker stimulation and systemic hyperoxia may be useful indexes for noninvasive monitoring of vascular function in retinas.

## Introduction

Tracking the status of retinal blood flow is important for investigating the pathogenesis of retinal vascular disorders, including diabetic retinopathy and branch retinal vein occlusion, but most studies on retinal blood flow have been cross-sectional. Basic information on how retinal blood flow changes with age in the same subject may help us to understand the longitudinal development of retinal diseases. Because the mice are commonly manipulated at genetic or systemic levels to investigate retinal diseases, it is important to have a noninvasive tool that allows reliable measurements of chronic change in retinal blood flow in such small size of eyeballs. Laser Doppler velocimetry (LDV), which enables the measurement of absolute values of retinal blood flow velocity, has been used for studies in cats^1^, pigs^2^, and humans^3^. However, this technology was not suitable for use in animals, such as mice and rats, with small eyeballs. The laser speckle flowgraphy (LSFG) is one of the newly developed methods used for quantitative estimation of ocular (optic nerve head, choroid, and retina) blood flow in both humans^4–6^ and animals^7,8^. In principle, LSFG assesses circulation in ocular tissues utilizing the laser speckle phenomenon, an interference event when coherent light sources, i.e., lasers, are scattered by a diffusing surface. Depending on the movement and velocity of blood cells, the speckle pattern varies rapidly and can be analyzed based on the space–time correlation function of the speckle intensity fluctuation^5^. The main outcome variables of LSFG are mean blur rate (MBR) in arbitrary unit, which is a quantitative index of blood velocity and has good correlation with blood flow measurements in various ocular tissues^5^. The flow velocity measured in vessels around and at the optic nerve head (ONH) appears to reflect the entire retinal circulation^9^.

Recently, the LSFG-Micro, a version of LSFG designed for small animals, has been implemented in rats by Wada et al.^8^ These investigators evaluated the reproducibility of LSFG-Micro measurements by characterizing ONH blood flow longitudinally. They found that ONH blood flow was gradually increased in rats from age 10 weeks to 19 weeks and then remained stable until age 60 weeks^8^. However, LSFG-Micro has not yet been validated for retinal blood flow measurement in mice, and we do not know whether similar longitudinal changes in retinal blood flow can be observed. Moreover, it is unclear whether LSFG-Micro can reliably detect retinal blood flow changes in response to local and systemic stimulations.

The increase in retinal blood flow in response to flicker light stimulation is an important index for retinal vascular function, which is coupled to the metabolic activity of the neural retina. Clinical studies have reported that flicker-induced dilation of retinal vessels is compromised in patients with diabetes^10^, glaucoma^11^, hypercholesterolemia^12^, and in regular smokers^13^. Moreover, systemic hyperoxia (inhalation of 100% oxygen) causes reduction of retinal blood flow, and this response was found to be blunted in diabetic patients associated with development of neovascularization in retinopathy^14^. Therefore, evaluating the time-dependent change in retinal blood flow in response to flicker stimulation and systemic hyperoxia may provide critical information on the development of retinal disease related to vascular disorders. However, the question remains unclear whether the responses of retinal blood flow to flicker stimulation and systemic hyperoxia, in relation to the function of neural retina (i.e., ERG), are altered longitudinally with age in the same subjects. To address these issues, we investigated and validated whether a LSFG-Micro can be used as a tool to consistently and reliably assess retinal circulation in response to flicker stimulation and systemic hyperoxia in mice. The longitudinal characterization of retinal blood flow was performed in mice from age 8 weeks to 20 weeks, the age that often used for biological research^15^.

## Results

### Longitudinal changes in systemic and ocular parameters

During the period of the experiments, significant increasing trends were found for the body weight, systemic blood pressure (SBP), diastolic BP (DBP), mean BP (MBP) and ocular perfusion pressure (OPP) from 8 to 20 weeks of age (Table 1). The intraocular pressure (IOP) was unaltered with age of the animals (Table 1).

**Table 1:** Longitudinal analysis of systemic and ocular parameters.

	Age, weeks							
	8	10	12	14	16	18	20	*P *value
Body weight (g)	23.6 ± 0.4	24.5 ± 0.2	25.2 ± 0.1	26.2 ± 0.2	26.8 ± 0.2	27.2 ± 0.2	27.8 ± 0.3	< 0.001
Systolic BP (mmHg)	74.2 ± 1.8	90.2 ± 3.7	84.1 ± 1.8	84.1 ± 3.0	86.0 ± 4.5	83.3 ± 1.4	99.0 ± 3.9	0.0011
Diastolic BP (mmHg)	45.3 ± 1.5	47.9 ± 3.8	55.5 ± 1.2	54.0 ± 1.3	56.2 ± 3.0	51.5 ± 0.9	60.4 ± 4.8	0.0064
Mean BP (mmHg)	55.0 ± 1.4	62.0 ± 2.9	65.0 ± 1.2	64.2 ± 1.6	66.3 ± 3.4	62.3 ± 1.0	73.3 ± 4.3	< 0.001
IOP (mmHg)	11.6 ± 1.0	10.5 ± 0.7	10.4 ± 0.3	10.5 ± 0.5	10.8 ± 0.8	10.5 ± 0.8	10.0 ± 1.0	1.0000
OPP (mmHg)	43.3 ± 1.4	51.5 ± 2.8	54.7 ± 1.3	53.7 ± 1.4	55.6 ± 3.1	51.8 ± 1.2	63.4 ± 4.9	< 0.001

During the period of the study, significant increasing trends were found for the body weight, systolic blood pressure (BP), diastolic BP, mean BP and ocular perfusion pressure (OPP) from 8 to 20 weeks old mice (n = 8). Jonckheere–Terpstra test, *P* < 0.01. Data are expressed as the mean ± SEM. IOP = intraocular pressure.

### Reproducibility of retinal blood flow measurement by LSFG-micro

**Figure 1:**
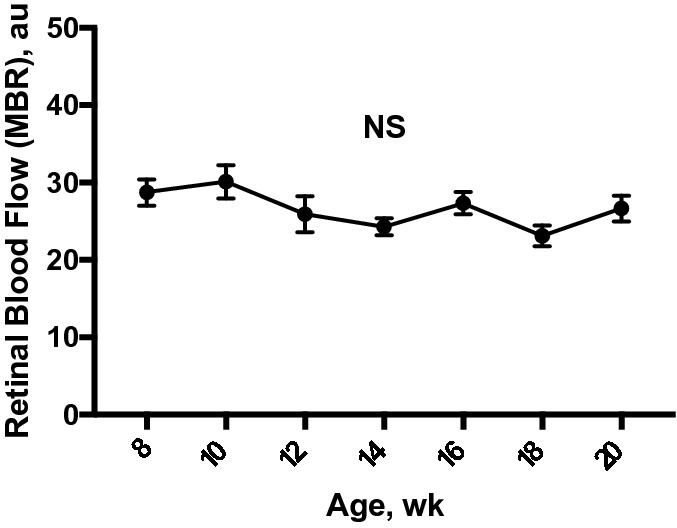
Time course of for the measurement of retinal blood flow (n = 8). There was no significant change in retinal blood flow from 8 to 20 weeks of age (*P* = 0.31*)* by repeated measures ANOVA. Data are expressed as the mean ± SEM.; au = arbitrary unit. NS = not significant.

There was no significant difference in the average baseline values of retinal blood flow measured by LSFG-Micro throughout the course of this study (n = 8; Fig. 1). The coefficient of variations (%) for the measurement of resting retinal blood flow every 2 weeks from 8 to 20 weeks were 2.8 ± 0.9%, 3.2 ± 0.6%, 3.8 ± 1.1%, 3.7 ± 1.0%, 3.5 ± 1.3%, 5.6 ± 0.4%, and 3.5 ± 0.6%, respectively, with no statistical difference.

### Assessment of retinal blood flow in response to hyperoxia

**Figure 2:**
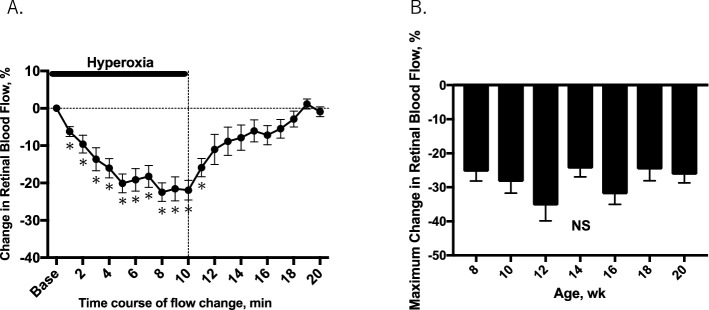
Time course of the percent changes in retinal blood flow from baseline in response to systemic hyperoxia in mice (n = 8) at 8 weeks of age (**A**). The mean peak change in retinal blood flow with time was calculated at different ages of the animal and presented as mean maximum change in retinal blood flow (**B**). Solid bar indicates the period of hyperoxia. *P < 0.05 compared with the baseline value by repeated measures ANOVA followed by the Dunnett’s test. Base = baseline; au = arbitrary unit; NS = not significant.

Retinal blood flow was decreased gradually then stabilized about 5 to 10 min after induction of systemic hyperoxia (100% oxygen; n = 8). The average maximum flow reduction was 25.5 ± 2.7% in 8-week old mice (Fig. 2A). After cessation of hyperoxia, retinal blood flow gradually returned to the baseline level within 10 min. The average maximum flow reduction by hyperoxia was not significantly different with age (Fig. 2B).

### Assessment of retinal blood flow in response to flicker stimulation

**Figure 3:**
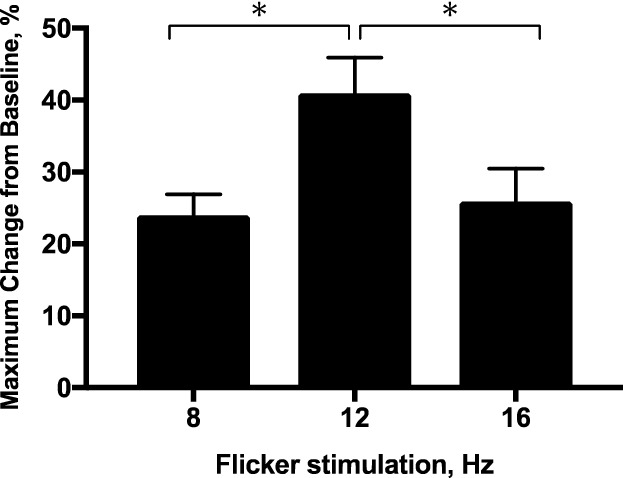
The maximum changes in retinal blood flow from baseline in response to flicker stimulation at 8, 12, and 16 Hz for 3 min in 8-week-old mice (n = 6). *P < 0.05 compared with 12-Hz by one-way repeated measures ANOVA followed by the Dunnett’s test.

In another series of studies, the optimal stimulation parameters of flickering light for flow response were determined in 8-week-old mice (n = 6). The peak changes in retinal blood flow in response to different frequencies (8, 12, and 16 Hz) of flickering light are shown in Fig. 3. With 12-Hz flicker stimulation, the increase in retinal blood flow (40.9 ± 5.0%) was higher than that produced by 8-Hz (24.0 ± 2.9%) and 16-Hz (25.9 ± 4.6%). Therefore, the 12-Hz stimulation was used for the subsequent experiments.

**Figure 4:**
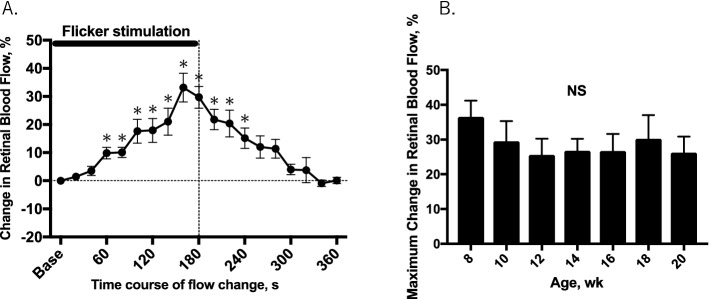
Time course of changes in retinal blood flow in response to 12-Hz flicker stimulation for 3 min in mice (n = 8) at 8 weeks of age (**A**). The mean peak change in retinal blood flow with time was analyzed at different ages of the animal and presented as mean maximum change in retinal blood flow (**B**). Solid bar indicates the period of 12-Hz flicker stimulation. *P < 0.05 compared with the baseline values by repeated measures ANOVA followed by the Dunnett’s test. Base = baseline; au = arbitrary unit; NS = not significant.

In response to flickering light, retinal blood flow increased with time and reached maximum level after 2 to 3 min of stimulation. The average maximum increase in retinal blood flow was 32.5 ± 5.0% in 8-week mice (n = 8; Fig. 4A). After cessation of the stimulation, retinal blood flow gradually returned to baseline within 3 min. The longitudinal experiments showed that the peak increase in retinal blood flow in response to 12-Hz flicker stimulation did not significantly change from age 8 weeks to age 20 weeks (Fig. 4B).

### Assessment of ERG parameters

**Figure 5:**
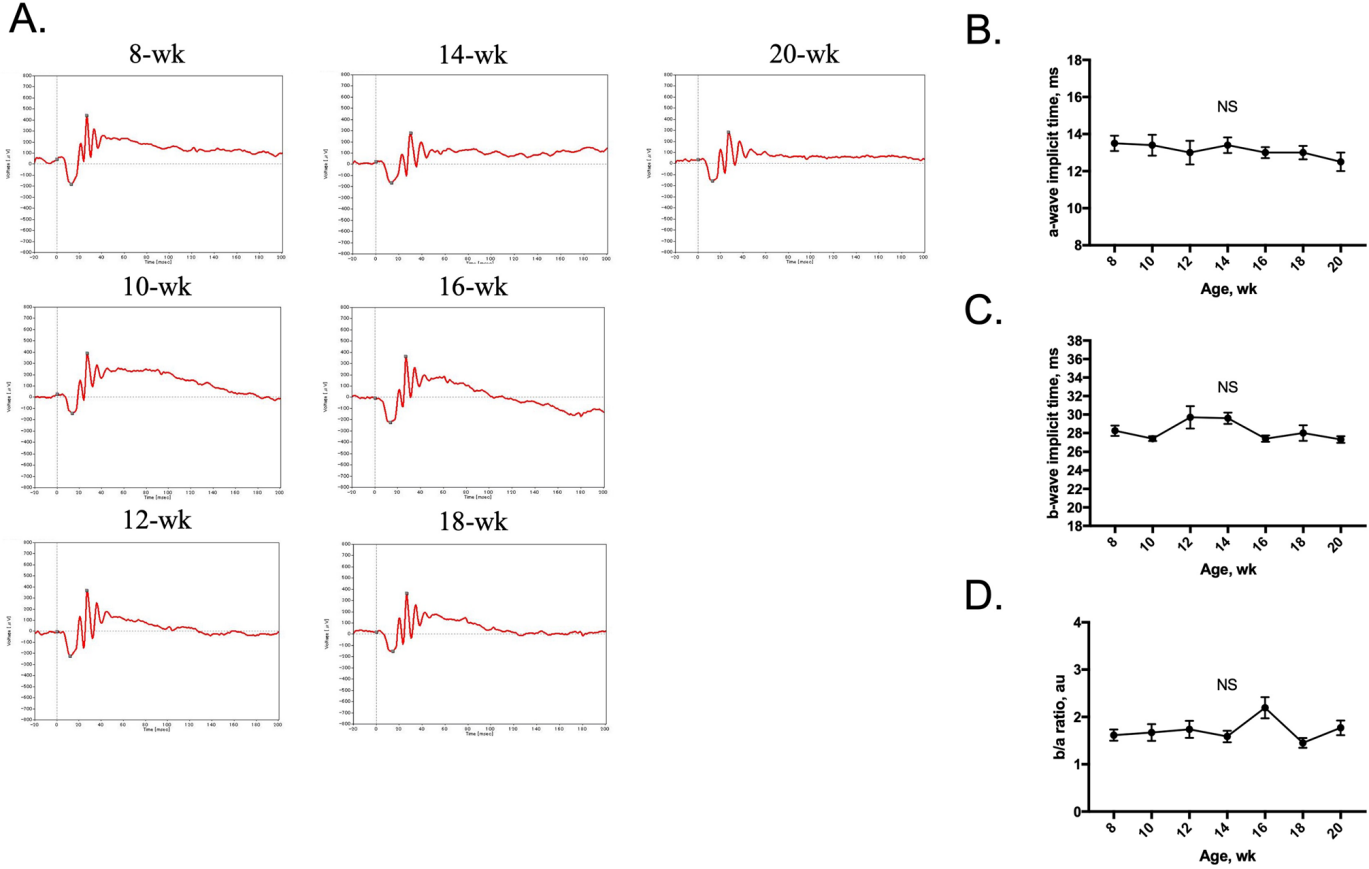
Representative ERG records obtained from the same animal from 8 to 20 weeks old (n = 8) (**A**). There were no longitudinal changes in a-wave implicit time (**B**) or b-wave implicit time (**C**) or the b/a ratio of the amplitude (**D**). au = arbitrary unit; NS = not significant.

The ERG was recorded under isoflurane anesthesia as the same condition as blood flow measurement experiment. The longitudinal assessment of ERG showed no significant changes in implicit times of a-wave and b-wave and the b/a ratio of the amplitude, along with the growth of the mice from 8 to 20 weeks old (Fig. 5).

## Discussion

In the current study, we found that the resting retinal blood flow remained relatively constant in mice from age 8 weeks to 20 weeks, whereas body weight and systemic BP increased significantly. In addition, the calculated OPP increased from 8 to 20 weeks of age. The reason for the dissociation of resting retinal blood flow from increased OPP during the growth of the animal is not currently understood. One possible explanation is that the oxygen extraction in the retinal circulation is increased with age, so the same amount of delivered blood remains sufficient for the need of the tissue. The development of flow regulation mechanisms with age, such as increase of myogenic tone of retinal vessels to adapt to the elevated perfusion pressure might also explain the unchanged retinal blood flow with increased OPP.

Wada et al., in a longitudinal study of ONH blood flow in rats, reported an increase of resting ONH blood flow from 10 to 19 weeks of age and then the flow was stabilized until 60 weeks, whereas the OPP remained unchanged from 10 to 60 weeks^8^. The reasons for these inconsistent results with ours are unclear. Although the study by Wada et al. differed from ours regarding species (rats vs. mice), anesthesia (pentobarbital vs. isoflurane), and interval of observations (10, 11, 13, 19, and 20 weeks, then every 5 weeks until 60 weeks of age vs. every 2 weeks until 20 weeks of age), further longitudinal studies are needed to address this issue. On the other hand, our results are consistent with a previous cross-sectional study showing no significant differences in retinal blood flow measured by LDV among healthy young, middle-aged, and elderly people^3^. Moreover, ERG parameters did not change from 8 to 20 weeks of age in our study, suggesting that there was no functional alteration in the neural retina which may indirectly support the observed consistency in retinal perfusion with age. In addition to ERG, the responses of retinal blood flow to flicker stimulation and systemic hyperoxia also remained unchanged throughout the course of the study (Figs. 2B, 4B). Taken together, our results demonstrate the homeostasis of the retinal circulation and the ability of the retina to maintain and regulate its blood flow in response to local (flicker) and systemic (hyperoxia) stimulations in mice, as the animals grow from 8 to 20 weeks old.

Small rodents, such as mice and rats, are widely used to study pathogenesis of acute and chronic eye diseases. Chaurasia et al.^16^ reported that retinal flow volume, measured with LSFG-Micro, was significantly decreased in diabetic mice at 6 months old. However, the reproducibility of the measurement and the longitudinal change of retinal blood flow in diabetes remain unknown. Moreover, the retinal flow volume reported in the aforementioned study^16^ was derived from the change of MBR in a single retinal vessel, which is difficult to extrapolate to the entire retinal circulation. In the current study, we were able to measure the MBR repetitively and reproducibly around and at the ONH. The MBRs were primarily collected, through software analysis, from conduit vessels originated from the central retinal artery and the retinal veins converged to the central retinal vein. The measurements of MBRs in the ONH area are considered to reflect the entire retinal circulation^9^.

In contrast to healthy subjects^17^, clinical studies have reported that the decrease in retinal blood flow in response to systemic hyperoxia (inhalation of pure oxygen) is blunted in patients with diabetes^18,19^. Diabetes also compromises the dilation of retinal vessels, as well as the increase of retinal blood flow, in response to flicker stimulation^20,21^. Although these cross-sectional studies have demonstrated the impairment of vasomotor function in diabetic retinas, the information on the time-dependent changes of retinal circulation along the course of disease development and progression cannot be drawn. Interestingly, a recent longitudinal study in mice found that alteration of ocular hemodynamics (both OPP and the flow velocity in central retinal artery) for 4 weeks could gradually exhaust the intrinsic ability of the retinal vessels to maintain stable resting blood flow^22^. However, this study did not evaluate the response of retinal blood flow to external stimulations. Our current study demonstrated the longitudinal stability of retinal vasomotor regulation in mice, from 8 to 20 weeks old, under resting conditions or subjected to external challenges. Although our results were derived from healthy animals, it would be interesting to investigate the longitudinal changes in retinal blood flow in diseased animals with the LSFG-Micro system. Nevertheless, our current study provides baseline information for future study of retinal blood flow dysregulation under disease states, including diabetes, hypertension and vascular remodeling that are known to impact retinal circulation.

The Dynamic Vessel Analyzer (DVA) has been used clinically^13,20,21,23^ to measure and analyze changes in diameters of retinal arterioles and venules in response to a 20- to 60-s flickering light (12.5 Hz) stimulation. In rats, the flicker response appears to occur within 15 to 60 s of stimulation^24,25^. Most recently, Albanna et al.^26^ reported the first study on vascular response to a 20-s flicker stimulation in mouse retina with DVA approach. They noted that there was no significant and reliable arterial reaction to flicker light in the majority of their measurements. On the other hand, the venous vessels responded with great heterogeneities both in types (i.e., dilation, no response, and constriction) and extent of the reactions^26^. In the present study, we employed a 180-s flicker stimulation and the retinal blood flow was found to increase at 60 s and then reached the maximum level at about 180 s (Fig. 4). It appears that a 20-s duration of flicker stimulation in the above mouse study^26^ might have been insufficient to elicit a consistent and noticeable retinal vasomotor response. The measurement of blood flow, i.e. fourth power amplification from caliber changes, in the present study is expected to be more sensitive and responsive for the evaluation of integrative vasomotor activity than the analysis of DVA from a single vessel. Recently, Fondi et al. reported the increase of ONH blood flow, measured by a LSFG, in response to flicker stimulation in humans^27^, and their results are comparable to our findings in mice.

In the current study, we found no longitudinal changes in a-wave and b-wave implicit times or the b/a amplitude ratio (Fig. 5). Previous studies have reported no age-dependent decline in ERG parameters in mice from age 3 months to 6 months^28,29^, and our results are consistent with those findings. Moreover, the observed normal neural function is in line with the unchanged resting retinal blood flow at different ages. The retinal perfusion appears to maintain neural retinal function as mice grow from 8 to 20 weeks of age.

The current study has some limitations. First, although we performed a longitudinal characterization of retinal blood flow in mice from 8 to 20 weeks old, the age of rodents often used for biological research^15^, the lifespan of mice can be up to 2 years. Therefore, further study with a longer follow-up period over one year is needed to address the effect of aging on retinal blood flow and neural function. Second, although we kept the level of isoflurane and the systemic parameters constant throughout the study, isoflurane per se can have effects on neural activity and might have impacted on our ERG results. However, a previous study reported that isoflurane anesthesia does not substantially alter the parameters of the murine ERG^30^, so anesthesia might only have had a little effect, if any at all in our study. Third, to reliably acquire retinal images and MBRs, administration of mydriatic agents is inevitable. However, the mydriatic eye-drop (0.5% tropicamide) administered in the present study might exert vasomotor activity in the retinal circulation and consequently confound our data interpretation. It is worth noting that topical application of commonly used mydriatic drugs (phenylephrine, tropicamide, cyclopentolate, or their combinations with 0.5–1.0% concentration) has no influence on macular blood flow^31^, retinal vascular diameters^32^ and microcirculation in healthy individuals^4^ or in glaucoma patients^33^. There are no differential impacts of mydriatic agents on retinal vascular reactivity and blood flow response to systemic hyperoxia in healthy individuals^34^. It appears that 0.5% tropicamide used in the present study is unlikely to exert significant impacts, if any, on the resting blood flow or vascular reactivity. Last, we measured blood flow in the region of ONH with an LSFG-Micro system, which does not allow arterioles to be distinguished from venules because of the tangled vascular network in the ONH area. Therefore, we were not able to examine arterioles and venules independently for blood flow regulation in the current study. Moreover, the recorded MBRs in the ONH area were mainly derived from the flow signal of red blood cells in the retinal vessels, but we cannot exclude the possibility of receiving blood flow signal from the choroid region or other vessels in the ONH. It is worth noting that retinal arterioles and venules may respond to systemic hyperoxia and/or flicker light differently in terms of their relative sensitivity and contribution to overall flow regulation. To further understand how the retinal blood flow is regulated at the microvascular level, a new technology is required to allow simultaneous assessment of temporal and special changes in arteriolar and venular blood flow, corresponding to the metabolic status of the surrounding neural tissue.

In conclusion, we found no apparent longitudinal change in resting retinal blood flow, neuroretinal function, and the blood flow responses to local (flicker light) and systemic (hyperoxia) stimulations as mice grow from 8 to 20 weeks old. The current study provides baseline information on the stability and dynamic changes in retinal blood flow under resting conditions and in response to external stimulations, respectively, in mice. Our results support the LSFG-Micro as a useful tool to longitudinally assess retinal blood flow in small animals.

## Materials and methods

### Animal preparation

We purchased 7-week-old C57BL/6J male mice (n = 14) from Charles River Laboratories JAPAN, Inc. (Yokohama, Japan) 1 week before the start of the experiment. The mice were housed in a temperature-controlled room with a 12-h dark and light cycle, with free access to food and water. The ethics committee of Nihon University approved the animal experiments, and the studies were conducted according to the tenets of the Association for Research in Vision and Ophthalmology. The animals were anesthetized with inhaled 2% isoflurane (Pfizer, Tokyo, Japan) under a constant flow rate of 1.5 L/min throughout the experiment. The rectal temperature was measured and maintained between 37 and 38 °C with a heated blanket. Pupils were dilated with 0.5% tropicamide (Santen Pharmaceutical Co., Osaka, Japan).

### Measurement of intraocular pressure and systemic blood pressure

Systemic blood pressure (BP) and intraocular pressure (IOP) were measured 30 min after induction of anesthesia. The systolic BP (SBP) and diastolic BP (DBP) were measured at the tail with an automatic sphygmomanometer (THC-31, Softron, Tokyo, Japan), and the IOP was measured with a handheld tonometer (TonolabTV02, ME Technical, Tokyo, Japan). The mean arterial BP (MABP) was calculated with the formula MABP = DBP + (SBP-DBP)/3. Because the mice were in a prone position during the experiments, the ocular perfusion pressure (OPP) was calculated with the formula OPP = MABP − IOP^35^.

### Measurement of retinal blood flow

**Figure 6:**
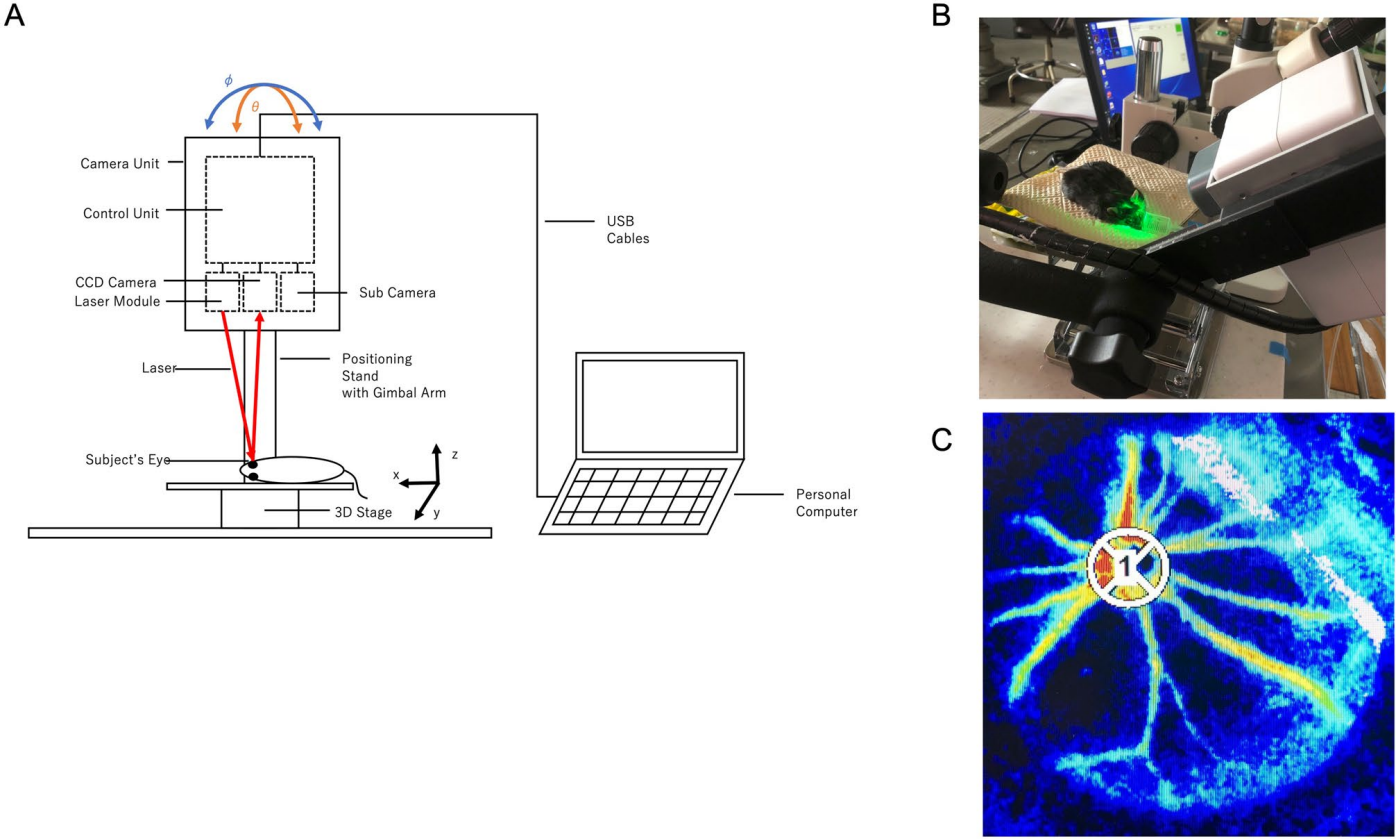
Schematic diagram and setup of the LSFG-Micro system. The LSFG-Micro system is set on a gimbal arm that allows transverse and lateral movements (orange and blue angles) so that a clear retinal image can be obtained (**A**). Experimental setup for measurement of retinal blood flow with LSFG-Micro in an anesthetized mouse (**B**). A representative color-coded map showing the circulation (MRB values) around the optic nerve head of a mouse. Red indicates a high MBR; and blue, a low MBR. A rubber o-ring (white circle) was placed over the region of the optic nerve head in the retinal image to confine the area for MBR measurement (**C**). 3D = three dimensional; CCD = charge-coupled device.

The mice were positioned on a stand with the right eye facing downward*.* A cover glass was placed on the left cornea with a drop of viscoelastic material, and retinal blood flow around ONH region in the left eye was measured with the LSFG-Micro system (Softcare Co., Ltd., Fukutsu, Japan). The LSFG-Micro system comprises a standard charge-coupled device (CCD) camera (700 × 480 pixels) equipped with a diode laser (830-nm wavelength) attached to a microscope (SZ61TR, Olympus Corporation, Tokyo, Japan) (Fig. 6A,B). The principle of LSFG-Micro is the same as that of LSFG, which was reported previously^8^. In brief, the mean blur rate (MBR), which represents a relative index of blood velocity, is determined by the blurring of the speckle pattern formed by the scattering of the laser by moving blood cells. The MBR images were acquired continuously at a rate of 30 frames/sec for 4 s. The field of view was 3.6 × 2.7 mm, with 53 mm working distance. Three consecutive measurements were performed without altering the position of the mice. The margin of the ONH was identified by manually placing a rubber o-ring (1.37-mm diameter) over the fundus image of the ONH (Fig. 6C). The vascular MBRs were derived from the subtraction of non-vascular (retinal tissue) MBRs from the total MBRs from the ONH area, calculated with LSFG analyzer software (version 3.2.19.0, Softcare Co., Ltd., Fukutsu, Japan). The MBR outputs were primary from conduit vessels originated from the central retinal artery and the venous vessels converged to the central retinal vein. The MBR measured in the vessels around and at the ONH appears to reflect the entire retinal circulation^9^. In the present study, the vascular MRBs were analyzed and referred as retinal blood flow as reported by other investigators^6,8,9,36^. The MBR outcomes were expressed as percentage change from the baseline.

### Induction of hyperoxia

Systemic hyperoxia was induced by inhalation of 100% oxygen for 10 min, as described in our previous study^37^. The mean of 3 flow measurements, obtained at 1-min interval for 3 min, was served as the baseline value before initiation of hyperoxia. The retinal blood flow was then measured every minute for 20 min during hyperoxia (10 min) and after termination of hyperoxia (10-min recovery).

### Flicker stimulation

The light source for the flicker condition was a white LED light. The light intensity for flicker stimulation was set at 30 lx for the rod-dominant mouse retina, in accordance with a previous study^26^. Before presentation of flicker stimuli, the ambient light was reduced to 1 lx or less. The mice were dark-adapted for 2 h and then adapted for a few minutes at the illuminance to be used for the experiment before flicker was initiated, as previously described^38,39^. The retinal blood flow was measured every 20 s for 6 min during (3 min) and after (3-min recovery) flicker stimulation. The mean of 3 measurements, obtained in 1 min at 20-s interval, was used as the baseline value before the initiation of flicker stimulation.

### ERG

The mice were dark-adapted for a minimum of 12 h before ERG was performed. They were transferred to a room with dim red light, and full-field ERGs were recorded with PuREC (Mayo, Inazawa, Japan) under systemic anesthesia with isoflurane. A ground electrode was placed at the tail, and a reference electrode was put in the mouth. Corneal electrodes were attached to the corneal surface bilaterally. To obtain the maximal response of both the cones and the rods, we used 3.0 cd s/m^2^ of flash. The amplitude of the a-wave was measured from baseline to the maximum a-wave peak, and the amplitude of the b-wave was measured from the nadir of the a-wave to the apex of the b-wave peak. The implicit times of the a- and b-waves were measured automatically by identifying the maximum negative and positive peaks on the ERG recording. The ratio of b-wave amplitude to a-wave amplitude (b/a ratio) was analyzed.

### Study protocol

To evaluate the reproducibility of the MRB measurement under resting conditions (i.e., baseline), we calculated the coefficient of variation (ratio of the standard deviation to the mean, expressed as a percentage) for 3 consecutive 5-min measurements of retinal blood flow every 2 weeks from age 8 weeks to 20 weeks of the animal. We performed the following assessments in each animal on 3 consecutive days every 2 weeks until the animals reached 20 weeks old: on day 1, the systemic hyperoxia was performed; on day 2, the flicker stimulation was performed; and on day 3, ERG was examined. A series of pilot studies was performed to confirm no significant changes in systemic BP, IOP, or OPP before, during, or after systemic hyperoxia or flicker stimulation. An independent masked observer performed all calculations and data analyses.

### Statistical analysis

All data are expressed as the mean ± SEM. The changes in retinal blood flow were calculated as percentage change from the baseline value. To analyze retinal blood flow responses to flicker stimulation and systemic hyperoxia, the peak change in retinal blood flow from baseline throughout the time course of flow measurement was identified and presented as maximum flow change in response to the stimulation. The Kolmogorov–Smirnov test was performed to assess the normality of data distribution. For statistical analysis, the Jonckheere–Terpstra test was used to evaluate the ordinal trend in the systemic and ocular parameters corresponding to the increase of animals’ age. A repeated-measured one-way analysis of variance (ANOVA) followed by the Dunnett’s test was used to determine the significance of experimental interventions, as appropriate. A *P* value < 0.05 was considered statistically significant.
